# Role of the *INDETERMINATE DOMAIN* Genes in Plants

**DOI:** 10.3390/ijms20092286

**Published:** 2019-05-09

**Authors:** Manu Kumar, Dung Thi Le, Seongbin Hwang, Pil Joon Seo, Hyun Uk Kim

**Affiliations:** 1 Department of Bioindustry and Bioresource Engineering, Plant Engineering Research Institute, Sejong University, Seoul 05006, Korea; manukumar@sejong.ac.kr (M.K.); dungcnshk55a@gmail.com (D.T.L.); sbhwang@sejong.ac.kr (S.H.); 2 Department of Chemistry, Seoul National University, Seoul 08826, Korea; pjseo1@snu.ac.kr

**Keywords:** indeterminate domain, sugar metabolism, flowering regulation, plant architecture, hormonal signaling, ammonium metabolism, stress signaling, seed development

## Abstract

The *INDETERMINATE DOMAIN (IDD)* genes comprise a conserved transcription factor family that regulates a variety of developmental and physiological processes in plants. Many recent studies have focused on the genetic characterization of IDD family members and revealed various biological functions, including modulation of sugar metabolism and floral transition, cold stress response, seed development, plant architecture, regulation of hormone signaling, and ammonium metabolism. In this review, we summarize the functions and working mechanisms of the *IDD* gene family in the regulatory network of metabolism and developmental processes.

## 1. Introduction

The Cys2His2 zinc-finger domain (C2H2) transcription factor family is one of the largest in plants. Analysis of 176 zinc finger proteins (ZFPs) from Arabidopsis revealed that 81% (143 ZFPs) were plant-specific (*Arabidopsis thaliana*, *Zea mays*, and *Solanum tuberosum*); only 19% (33 ZFPs) were conserved in other eukaryotes (Protozoa and animals) [1,2]. Extensive duplication has led to an expanded C2H2 family in plants [3]. Its subfamily *INDETERMINATE DOMAIN* (IDD), a conserved group across plants, contains functional genes that encode putative nuclear proteins with four zinc finger domains [4]. Since the report of the first plant *IDD* gene, *PCP1*, which is involved in sucrose uptake via an unknown mechanism in potatoes [5], many *IDD* genes have been functionally characterized. In general, IDDs form extensive protein interaction networks to ensure precise transcriptional control and thereby tissue- and/or cell-fate specification and hormonal signaling to control various aspects of plant growth and development [6,7,8,9].

Here, we provide a new understanding of the biological functions of *IDD* genes and their working mechanisms. We mainly focus on the role of IDDs in the linkage between sugar metabolism and developmental processes in plants.

## 2. Structure and Phylogenetic Analysis of IDD Proteins

**Figure 1:**
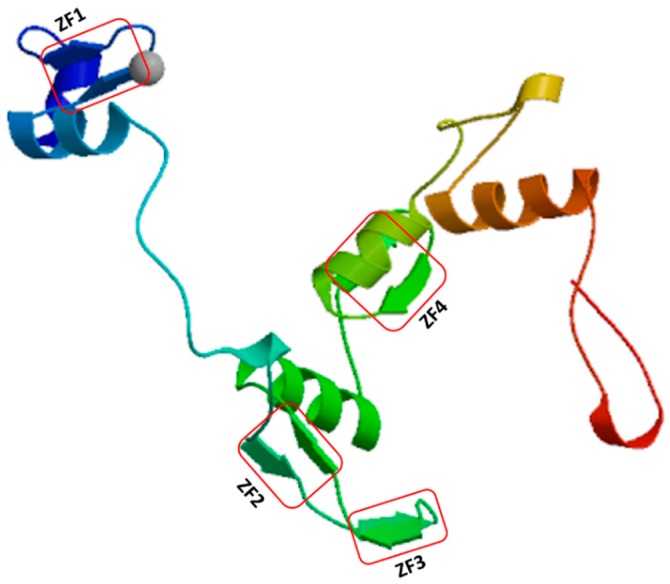
Predicted secondary structure of *Arabidopsis thaliana* AtIDD11 with functional zinc finger domains. It was generated by SWISS-MODEL (https://swissmodel.expasy.org/). The model predicts the monomeric protein chain binding to zinc atoms (grey circle). The red rectangles indicate the position of the four zinc finger motifs.

**Figure 2:**
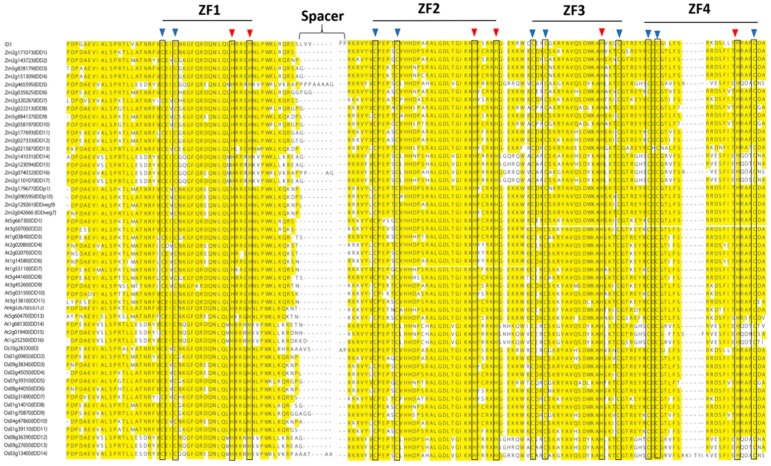
Comparative amino acid sequence alignment of *INDETERMINATE DOMAIN (IDD)* genes that shows motifs or domain that are conserved in different species. Alignment includes IDDs from *Arabidopsis thaliana* (AtIDD), *Oryza sativa* (OsIDD), and *Zea mays* (ZmIDD). Black boxes mark the position of cysteines (C, in blue triangles) and histidines (H, red triangles) characterized for each zinc finger.

**Figure 3:**
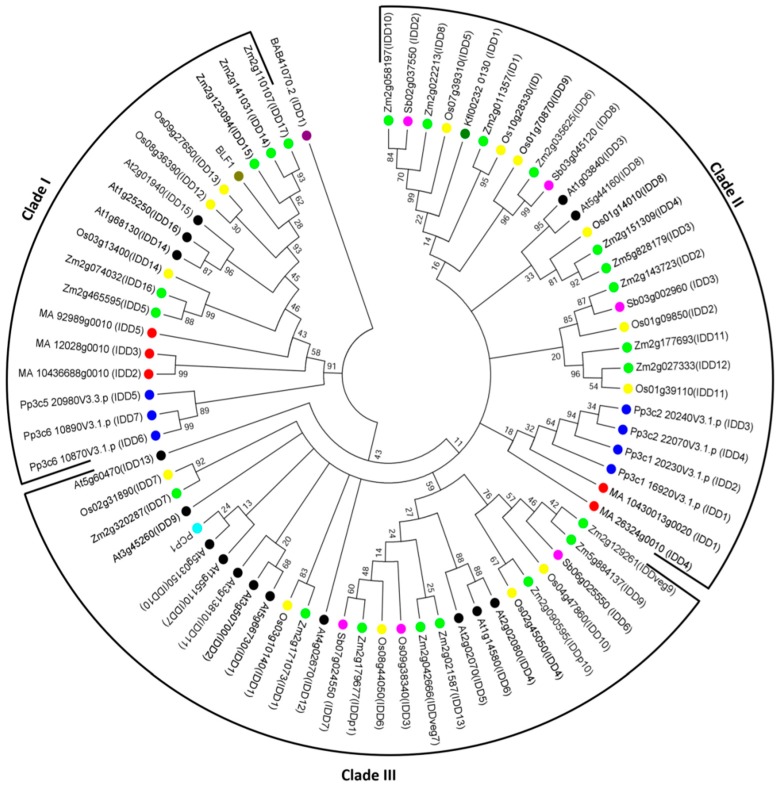
Phylogenetic analysis of IDDs from various plants generated using MEGA7 software. IDD amino acid sequences were collected by finding best hits using protein–protein BLAST at the NCBI [14], and from PlantTFDB database (http://planttfdb.cbi.pku.edu.cn/). The phylogenetic tree was made by using the neighbor-joining method, based on the JTT matrix-based model [15] with 1000 bootstrap replicates after amino acid sequences were aligned by Clustal W. Bootstrap values less than 10 were cut off. The tree is drawn to scale, with branch lengths measured in the number of substitutions per site. The phylogenetic tree includes 74 protein sequences with 17 dicot IDDs: 16 *Arabidopsis thaliana* (At, black), and 1 *Solanum tuberosum*, (Potato couch potato1 (PCP1), aqua); 43 monocots IDDs: 15 *Oryza sativa* (Os, yellow), 22 *Zea mays* (ID1 and ZmIDD, green), 5 *Sorghum bicolor* (Sb, fuchsia), and 1 *Hordeum vulgare* (BLF1, olive); 1 freshwater green algae IDD: *Klebsormidium flaccidum* (Kfl, teal); 5 Conifer IDDs: *Picea abies* (MA, red); 1 Fern IDD: *Ceratopteris reichardii* (BAB, purple), and 7 Moss IDDs: *Physcomitrella patens* (Pp, blue).

The *IDD* genes encode putative proteins containing four zinc finger motifs (ZF1-C2H2, ZF2-C2H2, ZF3-C2HC, and ZF4-C2HC) that bind zinc atoms, forming the core structure [4,10] (Figure 1). ZF1, ZF2, and ZF3 are important for DNA binding [11], whereas C2HC is required for RNA binding [12,13]. Amino acid sequence alignment showed that ZF1, ZF2, ZF3, and ZF4 motifs are conserved in many plant species (Figure 2 and Table S1). Evolutionary relationships of *IDD* genes in many plant species were investigated by constructing a phylogenetic tree using 74 IDD proteins from Arabidopsis (16), potato (1), maize (22), rice (15), barley (1), sorghum (5), conifers (5), ferns (1), mosses (7), and freshwater green algae (1). Three major clades were identified. Clade I included IDDs from mosses, conifers, maize, barley, rice, and Arabidopsis. Clade II contained IDDs of freshwater green algae, mosses, conifers, maize, rice, sorghum, and Arabidopsis. Clade III mainly contained IDDs identified as being from flowering plants, known as angiosperms, such as Arabidopsis, potato, maize, rice, and sorghum (Figure 3). This evolutionary relationship demonstrates the conserved biological function of IDDs in many plant species.

## 3. Biological Functions of IDDs

### 3.1. Modulation of Sugar Metabolism and Floral Transition

*ID1* regulates floral transition in maize [4,16,17]. Structural studies of ID1, along with other IDD proteins revealed the unique DNA-binding properties of two out of four zinc finger motifs [18], indicating that maize ID1 acts as a unique transcriptional regulator in the control of the floral transition. The *id1* mutant displays a prolonged vegetative phase without other developmental defects. In support, various genes involved in flowering significantly changed their expression in the *id1* mutant, including *ZCN8* and *ZMM4* [19,20]. The *ID1* ortholog in rice, *INDETERMINATE1* (*OsID1*)/*Early Heading Date2* (*Ehd2*), *Rice Indeterminate1* (*RID1*) is also involved in flowering [20,21,22,23]. *Ehd2* acts as a floral activator by upregulating *Ehd1* and the downstream floral activator genes, *Heading date 3a* (*Hd3a*) and *Rft1* (*Hd3b*) genes in a unique regulatory network of photoperiodic flowering [20,21,22,23].

As a source of energy and carbon, sugar is the most important nutrient for growth and development of nearly all living organisms. Sugar metabolism is most likely associated with the floral transition, and IDDs are core members involved in the crosstalk. *PCP1*, an *IDD* gene of *Solanum tuberosum*, was shown to activate the silent endogenous sucrose uptake system in yeast [5]. The yeast strain SUSY7, which lacks an endogenous invertase gene, is unable to grow on sucrose-containing medium, but can be rescued by complementation of *PCP1*. Moreover, expression of *PCP1* also rescues the yeast mutant strains that have defects in sucrose synthase and sucrose transport, although the underlying molecular mechanism remains unclear [5].

**Figure 4:**
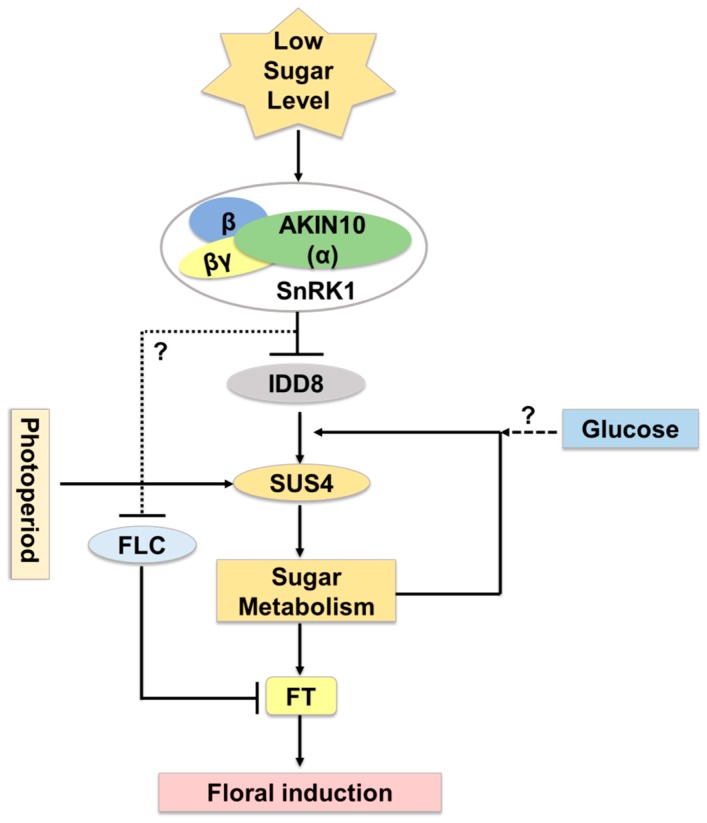
*AtIDD8-SUS4* module functioning in flowering time regulation in *Arabidopsis thaliana*. Under sugar deprived conditions, AKIN10, the α catalytic subunit of SnRK1 kinase, blocks IDD8 activity. IDD8 is phosphorylated at ser-178 and ser-182 positions to decrease its transcriptional activation activity, and thus, consequently, *SUS4* expression. Additionally, endogenous sugar levels give feedback regulation to control the expression of *SUS4*. *AKIN10* also has a role in the negative regulation of FLOWERING LOCUS C (FLC), which acts to suppress *FLOWERING LOCUS T* (*FT*), a floral activator in Arabidopsis.

Several Arabidopsis IDD members function as transcriptional regulators of floral transition, possibly through the control of sucrose signaling [24]. In Arabidopsis, *AtIDD8* has been reported to function in sugar metabolism and contribute to photoperiodic flowering [25]. Expression of Sucrose Transporter genes (*SUC2*, *SUC6*, *SUC7*, and *SUC8*) and Sucrose Synthase genes (*SUS1* and *SUS4*) are affected by *IDD8* activity. IDD8-SUS4 module-regulated sugar metabolism is associated with photoperiod flowering [25,26]. AtIDD8 is further regulated through phosphorylation at two positions, Ser-178 and Ser-182, which is catalyzed by the catalytic α-subunit of Sucrose-non-fermenting1 (Snf1)-related kinase 1 (SnRK1)/AKIN10 [27]. Phosphorylation of AtIDD8 significantly reduced its transcriptional activation activity [27]. Consistently, *atidd8* mutants and plants overexpressing *AKIN10* display a delayed-flowering phenotype. This pathway can be referred to as a gatekeeping mechanism for plants to regulate floral transition in a malnourished, low sugar level state (Figure 4).

**Figure 5:**
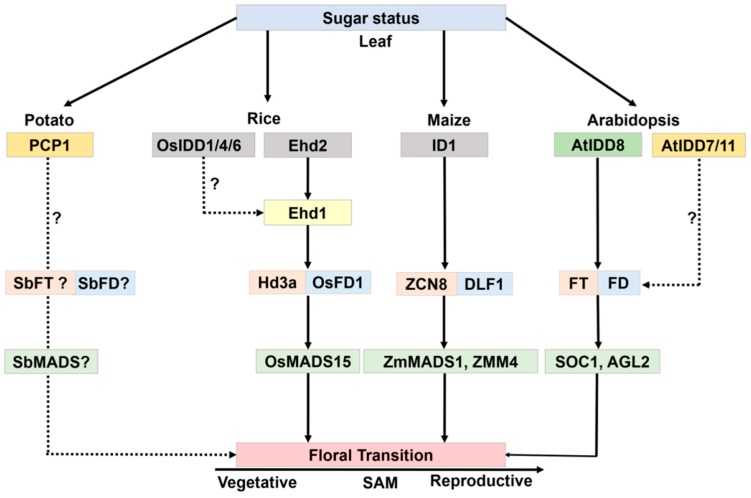
Schematic representation of IDD members that might be involved in sugar transport and floral transition in the plant. Same color boxes are orthologous *IDD* genes. Dotted lines represent an unknown pathway. Genes with a question mark are still to be studied for floral transition.

At the pre-flowering stage, maize *id1* mutant leaves have a significantly lower ratio of sucrose formation from starch [28]. Variations in sucrose and starch levels in *id1* suggest an *ID1* role in promoting carbohydrate export to the shoot apex upon flowering [28]. Another study reported the involvement of the sugar signaling molecule trehalose 6-phosphate (T6P) in developmental growth, including flowering [29]. Phloem-specific induction of the Arabidopsis florigen *FLOWERING LOCUS T* (*FT*) can rescue lines that have a late-flowering phenotype because of reduced expression of *TREHALOSE-6-PHOSPHATE SYNTHASE1* (*TPS1*) [30,31]. These results indicate that sugar status linked to T6P signaling is vital for flowering [31]. In rice, *OsIDD1* and *OsIDD6* overexpression rescue the late-flowering phenotype of rice *Ehd2*, illustrating that IDD family genes might have a functional redundancy in sugar metabolism and the control of flowering time [32]. These reports suggest that multiple *IDD* genes modulate sugar metabolism, and some of them have a direct or indirect link with flowering regulation (Figure 5).

### 3.2. Starch Accumulation and Cold Response

Abiotic stress, including salinity, drought, and cold, adversely affects plant growth and development. Cold stress is a significant environmental challenge, and plants have evolved various strategies to ensure plant fitness [33]. In Arabidopsis, two splice variants of *AtIDD14* (*AtIDD14α* and *β*) directly regulate starch metabolism in response to cold via regulation of *Qua-quine starch* (*QQS*) expression. *QQS* represses starch accumulation [34]. The functional *AtIDD14* form (*AtIDD14α*) binds to the *QQS* promoter and activates its expression, promoting starch degradation. The non-functional *AtIDD14β* form, which is produced mainly under cold conditions (4 °C), lacks a functional DNA-binding domain, but can form a heterodimer complex with *AtIDD14α*. Thus, the *AtIDD14β* isoform acts as a competitive inhibitor to repress DNA binding activity of *AtIDD14α*. Therefore, *QQS* is repressed by cold stress via the self-regulatory module provided by cold-induced alternative splicing. Competitive inhibition of *AtIDD14α* activity by *AtIDD14β* would serve as a cold adaptation strategy, helping plants maintain an appropriate level of starch accumulation during the dark period; this might be required to tolerate low temperatures during the light period [35].

In rice, an *IDD* gene encoding the ROC1 protein binds the *CBF1* promoter directly to regulate cold tolerance [36]. Transcription activator *CBF1* contains an AP2 domain, and it controls many cold-responsive genes [37]. In rice, *MYB15* also controls the expression of *CBF1* gene in cold stress. These results indicate that there might be some complex system that helps *CBF1* regulate cold stress response, along with *ROC1* and *MYB15* [36,38,39,40]. The function of IDD in low temperature is remarkable. However, which endogenous signal activates *ROC1* or *AtIDD14* is less understood. Previous papers have shown that cold stress induces the alternative intracellular auxin gradient via auxin transporter gene (*YUC*, *PIN*) to regulate plant growth and development. Auxin and other phytohormones-responsive genes also respond to cold stress [41]. On the other hand, both *AtIDD14* and *ROC1* are reported to be involved in auxin signaling [6,36]. This suggests that auxin might induce *IDD* activity under cold-stress conditions.

### 3.3. Regulation of Seed Development

Seed development and maturation is a crucial process in the life cycle of a plant. IDDs are involved in the regulation of seed development. In maize, duplicated genes *ZmIDDveg9*/*NAKED ENDOSPERM (NKD1*) and *ZmIDD9*/*NKD2* are involved in seed maturation, cell differentiation, thick walls, and accumulation of anthocyanin pigments [42]. These genes are required for aleurone cell fate and cell differentiation. Genetic mutations of the *IDD* genes *Zmiddveg9* and *Zmidd9* lead to naked endosperm phenotypes, decreases in germination rates, starch accumulation, delayed anthesis, less seed weight, and a propensity for vivipary [42,43]. NKD1 and NKD2 can directly regulate transcription and activate *viviparous1* and *opaque2* genes. Further, NKD2 functions as a negative regulator of NKD1 [42,43].

In Arabidopsis, *AtIDD1* acts as either an activator or a repressor of germination, depending on the absence or presence of gibberellic acid, respectively. *GID1b* encoding a GA receptor is the target of GAF1/AtIDD1. Ectopic expression of *IDD1*/*ENY* under CaMV35S leads to disrupted seed development, delayed endosperm depletion, testa senescence, and an impaired maturation program. Subsequently, mature *2x35S:ENY* seeds have high endosperm-specific fatty acids, starch retention, and defective mucilage extrusion with low expression of *GID1b* [4,5]. Studying the molecular mechanisms of IDD function, including transcriptional regulation of downstream gene networks, will provide a better understanding of regulated seed development, and the knowledge attained can be expanded to important work on cereal grain quality.

### 3.4. Modulation of Plant Architecture, Shoot Gravitropism, and Secondary Cell Wall Formation

Plant architecture influences plant fitness and productivity. IDDs play a role in organ development, and thereby plant architecture. In rice, secondary cell wall formation is negatively regulated by *OsIDD2* [44]. Transgenic plants overexpressing *OsIDD2* display dwarfism, fragile leaves, and decreased lignin content [45], whereas an *osidd2* knockdown mutant produced by the CRISPR/Cas9 technique showed high lignin content. In particular, *OsIDD2* downregulates genes involved in lignin biosynthesis and sucrose metabolism [44]. The *Loose Plant Architecture1* (*LPA1*) gene, a functional ortholog of *AtIDD15*/*SGR5* in rice, also affects the plant architecture, especially shoot gravitropism [46,47]. The *lpa1* mutant coleoptile exhibits slower sedimentation rate of amyloplasts compared to wild-type [47]. The coleoptile of the *lpa1* mutant exhibits negative gravitropism, indicating that signal transduction or gravity sensing is disturbed in the mutant. *LPA1* also blocks auxin signaling through its interaction with C-22-hydroxylated and 6-deoxo brassinosteroids (BRs), which in turn regulate lamina inclination [46,47]. *lpa1* mutants display indole-3-acetic acid (IAA) hypersensitivity during the lamina inclination response, which can be suppressed by brassinazole (Brz) (an inhibitor of C-22 hydroxylase involved in BR synthesis). Roles of *LPA1* in *OsPIN* gene expression (*OsPIN1a*, *OsPIN1c*, and *OsPIN3a*) further indicate that the *LPA1*-mediated lamina inclination in rice might be due to auxin flux [46,47].

In barley, IDD protein BLF1 acts as a regulator of the leaf-width growth [48]. The *blf1-1* mutation leads to wider but slightly shorter leaves than wild-type, because of a perturbation in the longitudinal cell numbers in leaves. A BLF1-vYFP fusion protein indicates *BLF1* expression in the shoot apical meristem, epidermis, and prospective veins of leaf primordia. Given the economic and agronomical value of leaf traits in crop plants [49,50], *BLF1* might be an ideal candidate for optimizing crop architecture.

In Arabidopsis, some *IDD* genes are associated with cellular patterning. Among them, *AtIDD14-A* (a spliced variant of *AtIDD14*), *AtIDD15*, and *AtIDD16* regulate lateral organ morphogenesis and gravitropism by promoting auxin biosynthesis and transport [6]. Since IDDs are also involved in starch metabolism, coordination between auxin accumulation and starch metabolism may underlie plant development. For example, the zinc finger transcription factor, *SHOOT GRAVITROPISM5* (*SGR5*)/*AtIDD15*, has a crucial role in the early events of gravitropic responses in the inflorescence. The *SGR5* gene has two splice variants: the truncated *SGR5β* form that lacks the functional ZF motifs and the full-size *SGR5α* transcription factor [51]. A truncated form of SGR5β inhibits SGR5α function, possibly by forming non-functional complex heterodimers. High temperatures might accelerate the alternative splicing of *SGR5*, resulting in a high level of accumulation of SGR5β proteins. *SGR5β* over-expression plants exhibit reduced gravitropic response of the inflorescence stem, similar to that of the *atsgr5-1* phenotype [52].

**Figure 6:**
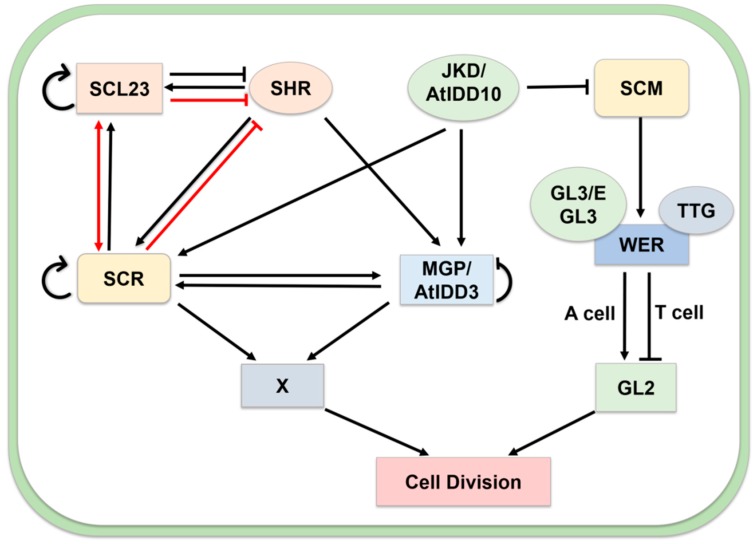
Role of the MGP/AtIDD3 and JKD/AtIDD10 in cell division. JKD regulates the formation cells through two pathways: (1) Complex SHR-MGP-SCR-JKD with putative target (X) genes. SHR enhances expression of SCL23 and SCR and MGP. SHR negatively regulated via protein–protein interaction to SCL23 and SCR. Both SCR, MGP, and SCL23 can self-control their transcription. JKD directly regulates SCR and MGP expression in cooperation with MGP, SCR, and SHR. (2) JKD modulates the repression of SCM that leads to the activity of the GL3/EGL3/TTG1 complex, which depends on the relative abundance of an MYB transcription factor, WEREWOLF (WER). WER triggers the trichoblasts (T cell) to inhibit GL2 and atrichoblasts (A cell) to lead cell division. Arrows and bars represent positive regulation and negative regulation, respectively. Transcriptional controls are depicted with black arrows, protein–protein interactions are described with red arrows. The distinction of box color and shape describes different genes in this network.

JACKDAW (JKD/AtIDD10) and MAGPIE (MGP/AtIDD3) modulate the expression of SHORT-ROOT (SHR) and SCARECROW (SCR) in the root apex [53,54,55] (Figure 6). SCR and SHR are two GRAS family transcription factors, which are required for quiescent center and ground tissue formation in roots. JKD directly regulates *SCR* and *MGP* expression in cooperation with SHR [54]. SHR is a crucial regulator that directly activates the expression of *SCL23* and *SCR*. In the SHR-SCR-SCL23 complex, SHR level is modulated by SCL23. The SHR-SCR-SCL23 complex plays a crucial role in endodermis formation in the hypocotyl [56]. JKD also modulates the repression of *SCM* that leads to the activity of the GLABRA3 (GL3)/ENHANCER OF GLABRA3 (EGL3)/TRANSPARENT TESTA GLABRA1 (TTG1) complex. This complex depends on the relative abundance of an MYB transcription factor, WEREWOLF (WER). WER triggers the trichoblasts (T cell) to inhibit GL2 and atrichoblasts (A cell) to lead cell division [53,57].

### 3.5. Regulation of Hormonal Signaling

Hormone signaling has diverse and crucial roles in plant development. Hormone interactions control the formation of all organs in the plant by regulating meristem function. Gibberellins (GA), auxin, cytokinin (CK), brassinosteroids (BRs), and strigolactones (SLs) play vital roles during plant development, from embryogenesis to senescence [58]. DELLAs, the GRAS transcriptional regulators containing a GRAS domain at the C terminus and a DELLA/TVHYNP motif at the N terminus [9], act as key players in the regulation of GA responses. They lack a DNA binding domain, and act as transcriptional coregulators with other DNA-binding factors. Notably, five IDD members, AtIDD3, AtIDD4, AtIDD5, AtIDD9, and AtIDD10, interact with DELLA and regulate the GA-positive regulator, *SCARECROW-LIKE3* (*SCL3*) [8,9,59,60]. Further experiments have indicated that DELLA and SCL3 act as coregulators and require IDDs as transcriptional scaffolds for DNA binding to check GA feedback regulation. IDD binding to DNA represents the balance of the SCL3 and DELLA protein levels to regulate downstream gene expression.

Auxin is another essential plant hormone that has a crucial role in controlling plant development processes, including embryogenesis, gametogenesis, patterning, lateral organ formation, tropic responses, and branching [61,62]. Auxin-mediated developmental and growth events are shaped by auxin biosynthesis and intercellular polar transport [61,63]. Some IDDs regulate auxin biosynthesis and transport. The epinastic leaves in plants overexpressing IDDs (*IDD14*, *IDD15*, and *IDD16*) are similar to those in auxin overproduction plants [6,64,65,66,67]. The IDD proteins directly bind to the promoter regions of *TAA1*, *PIN1*, and *YUC5* and activate their expression. *IDD*-regulated auxin signaling might be further regulated by *ZAT6* [68]. Further study of crosstalk between hormone metabolism and the surrounding environment will lead to a better understanding of the role of IDD proteins in the regulation of hormonal signaling.

### 3.6. Ammonium Metabolism

In the roots of higher plants, ammonium and nitrates are the primary sources of nitrogen (N). Asparagine and glutamine are the primary forms of organic N, and are transported to the shoots from the roots via the xylem [69]. Many reports have suggested a possible role of N in the various developmental and metabolic processes [70,71,72,73,74]. In rice, *OsIDD10* directly activates the transcription of *AMT1*;*2* (ammonium transporter) and *GDH2* (glutamate dehydrogenase, which degrades glutamate to ammonia and alpha-ketoglutarate). Further, *OsIDD10* also upregulates genes involved in N-linked metabolism, including nitrite reductases, glutamine synthetase 2, and trehalose 6-phosphate synthase [10,75]. Notably, *OsIDD10* plays an essential role in the interaction between NH_4_^+^ and auxin signaling in rice roots [76]. The gravity response was delayed in *osidd10* roots and accelerated in *OsIDD10* overexpression (*IDD10*-OX) roots in the absence and presence of NH_4_^+^, respectively [69,76]. However, treatment with 1-N-naphthylphthalamic acid (NPA), a polar auxin transport inhibitor, suppressed the NH_4_^+^-induced root specific phenotype of the *osidd10*. The expression of NH_4_^+^-mediated auxin-related genes is affected in *osidd10* and *OsIDD10* overexpression plants. Phenotypes and expression patterns triggered by NH_4_^+^ are influenced by the actions of auxin during root development, suggesting a regulatory circuit in rice between NH_4_^+^ and auxin signaling that functions in root development [76]. The fact that *IDD10* induces the expression of genes for trehalose-6-phosphate synthase, aminotransferase, and cytokinin dehydrogenase further strengthens the possibility of functional involvement of the gene in N-linked metabolism [10]. Therefore, it will be essential to perform a metabolite analysis to determine the possible agricultural benefits of manipulating *IDD10* to enhance the efficiency of N metabolism in crop plants.

## 4. Conclusions

**Figure 7:**
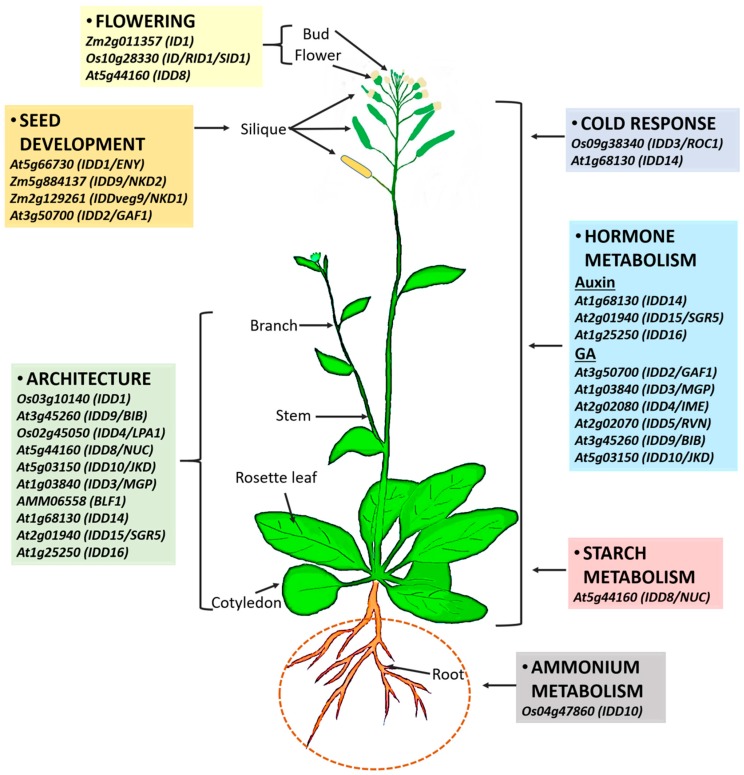
Overview of multiple functions of IDDs in plant growth and development based on previous reports. IDDs control flowering transition, regulate seed development, metabolism of starch, hormones, and ammonium, and are involved in responsiveness to cold stress.

The IDD protein family comprises plant-specific transcription factors that have primary functions in inflorescence, leaf architecture, root architecture, seed development, and sugar homeostasis [77]. Most *IDD* genes have been mainly characterized in Arabidopsis; however, a few have been functionally characterized in other plants (Table 1). They are involved in seed maturation and germination, GA signaling, root development, sugar metabolism, leaf polarity, starch metabolism, cold-stress signaling, auxin biosynthesis and transport, flowering, plant architecture, shoot gravitropism, ammonium uptake, and endosperm development (Figure 7) [4,5,7,8,9,25,32,36,42,43,44,47,48,53,54,55,75,77,78,79,80,81,82]. IDD activity regulates many traits that have a direct or indirect impact on crop yield. Specifically, traits such as leaf angle contribute to overall plant architecture. Sugar metabolism and flowering time contribute to the allocation of carbon and grain yield, and endosperm development to seed maturation. Based on the fact that IDDs govern nearly all aspects of plant growth and development, information about IDDs will provide invaluable insights into the genetic programs underlying signaling networks in the regulation of plant development and metabolism with connections to external environmental fluctuations.

## Figures and Tables

**Table 1:** List of *IDD* genes described in this study.

Gene Name	Phenotype	Key Role	References
Arabidopsis			
*At5g66730* (*AtIDD1*)/ENHYDROUS	Overexpression of *AtIDD1* increases starch retention, endosperm-specific fatty acids, and defective mucilage extrusion in mature seed.	Seed development	[7]
*At3g50700* (*AtIDD2*)/CARRION CROW/*GAF1*	*idd2/idd1* double mutant exhibit decreased GA responsiveness, whereas *IDD2* overexpressor enhance GA responsiveness.	GA signaling	[8]
*At1g03840* (*AtIDD3*)/MAGPIE	SHORT-ROOT (SHR) transcription factor directly targets *AtIDD3/MGP*, which plays key roles in specifying the radial root patterning and root stem cell niche.	Root development, DELLA interacting protein	[9,83]
*At2g02080* (*AtIDD4*)/IMPERIAL EAGLE	IDD4 and GR-REV act together to coordinate and promote outgrowth of a flattened leaf blade. IDD4 is an SHR target.	DELLA interacting protein, leaf polarity	[79,81]
*At2g02070* (AtIDD5)/RAVEN	Positive regulator of starch synthase 4. *atidd5* mutants have deformed chloroplasts and starch granules.	DELLA interacting protein	[84]
*At1g14580* (*AtIDD6*)/BLUEJAY	Stem cell regulator and participant in root initiation, and is required to pattern new organs.	Root development	[79,85]
*At1g55110* (*AtIDD7*)/REDSTART	N/R		
*At5g44160* (*AtIDD8*)/NUTCRACKER	*idd8* mutants exhibit delayed flowering under LD condition.	Control floral transition via modulation of sugar metabolism, root development	[25,27,79,86]
*At3g45260* (*AtIDD9*)/BALDIBIS (Cabib and Leloir)	Jointly stabilizes tissue boundaries by confining the cell fate regulator SHORT-ROOT and contributing to fate specification.	Restrict SHR movement in root tissues	[87]
*At5g03150* (*AtIDD10*)/JACKDAW	JKD physically interacts with cell fate determinants SHR and SCR in a cell-type-specific manner.	Root development, DELLA interacting protein	[9,53,54,55,88]
*At3g13810* (*AtIDD11*)/WARBLER	N/R	Leaf polarity (regulated by REV and KAN1)	[81]
*At4g02670* (*AtIDD12*)/WOODPECKER	N/R		
*At5g60470* (*AtIDD13*)/EGRET	N/R		
*At1g68130* (*AtIDD14*)	*idd14-1* mutant shows diverse leaf phenotypes. 35S*: IDD14*α has retarded growth and downward leaf curling (same 35S: QQS), whereas 35S: *IDD14β* and *idd14-1* mutants show slightly early flowering.	Auxin biosynthesis and transport, starch metabolism in response to cold stress	[6,35]
*At2g01940* (*AtIDD15*)/SGR5	*idd15-5* increases angles between inflorescence stem or branches and siliques.	Auxin biosynthesis and transport	[6,80,82]
*At1g25250* (*AtIDD16*)/FALCON	IDD16-RNAi transgenic plant has the same phenotype as *idd15-5*. *idd14-1* and IDD16-RNAi transgenic plants have enlarged floral organs and infertile siliques.	Auxin biosynthesis and transport	[6]
Rice			
*LOC_Os10g28330* (*OsID*)/*SID1*/*RID1*	*rid1* causes the never-flowering phenotype and gain of function of *SID1*, *OsIDD1* or *OsIDD6* restore the *rid1* phenotype.	Flowering transition	[23,32]
*LOC_Os01g09850* (*OsIDD2*)	*OsIDD2*-overexpression plants showed severe dwarfism with height reaching about half that of the wild-type plants, whereas *OsIDD2*-RNAi and *osidd2* rescued the phenotype.	Secondary cell wall structure	[44]
*LOC_Os09g38340* (*OsIDD3*)/*ROC1*	*roc1* mutant shows hypersensitivity to chilling stress.	Cold response	[36]
*LOC_Os03g13400* (*OsIDD14*)/*LPA1*	*lpa1* mutant causes loose plant architecture, reduces shoot gravitropism.	Shoot gravitropism	[47]
*LOC_Os04g47860* (*OsIDD10*)	*idd10* mutant roots show hypersensitive to exogenous ammonium	Ammonium uptake and nitrogen metabolism	[10,75,76]
*Zea mays*			
*Zm2g011357* (*ZmID1*)	*id1* mutant did not undergo a healthy transition to flowering and remained in a prolonged vegetative growth.	Flowering transition	[4,89,90]
*Zm2g129261* (*ZmIDDveg9*)/*NKD1* *Zm5g884137* (*ZmIDD9*)/*NKD2*	*nkd1* and *nkd2* mutants are important for differentiation, cell patterning, seed maturation, and resource reserve deposition	Endosperm development	[9,43,91]
Domesticated barley			
AMM06558 (*DbBLF1*)	Overexpression of *LEAF1* reduced leaf width, *leaf1-1* mutant was in contrast.	Regulate barley leaf size	[48]
*Solanum tuberosum*			
*X82328* (*StPCP1*)		Activate uptake of endogenous sucrose	[5]

* N/R: Not yet reported.

## Supplementary Materials

The following are available online at https://www.mdpi.com/1422-0067/20/9/2286/s1.

## Abbreviations

IDD	INDETERMINATE DOMAIN
LPA	Loose Plant Architecture
TPS	Trehalose-6-phosphate synthase
FT	Flowering Locus T
RID	Rice Indeterminate
Ehd	Early heading date
SUS	Sucrose Synthase
BLF	Broadleaf
AMT	Ammonium Transporter
NKD	Naked Endosperm
ENY	Enhydrous
HD	Heading Date
Rft1	Rice Flowering Locus T1
